# Review: Unravelling the Role of DNA Sensing in Alum Adjuvant Activity

**DOI:** 10.1093/discim/kyac012

**Published:** 2022-12-29

**Authors:** Zara Gatt, Utku Gunes, Arianna Raponi, Larissa Camargo da Rosa, James M Brewer

**Affiliations:** School of Infection & Immunity, College of Medical, Veterinary and Life Sciences, University of Glasgow, Scotland; School of Infection & Immunity, College of Medical, Veterinary and Life Sciences, University of Glasgow, Scotland; School of Infection & Immunity, College of Medical, Veterinary and Life Sciences, University of Glasgow, Scotland; School of Infection & Immunity, College of Medical, Veterinary and Life Sciences, University of Glasgow, Scotland; School of Infection & Immunity, College of Medical, Veterinary and Life Sciences, University of Glasgow, Scotland

**Keywords:** adjuvants, alum, vaccines, mechanism, DNA sensing

## Abstract

Public interest in vaccines is at an all-time high following the SARS-CoV-2 global pandemic. Currently, over 6 billion doses of various vaccines are administered globally each year. Most of these vaccines contain Aluminium-based adjuvants (alum), which have been known and used for almost 100 years to enhance vaccine immunogenicity. However, despite the historical use and importance of alum, we still do not have a complete understanding of how alum works to drive vaccine immunogenicity. In this article, we critically review studies investigating the mechanisms of action of alum adjuvants, highlighting some of the misconceptions and controversies within the area. Although we have emerged with a clearer understanding of how this ubiquitous adjuvant works, we have also highlighted some of the outstanding questions in the field. While these may seem mainly of academic interest, developing a more complete understanding of these mechanisms has the potential to rationally modify and improve the immune response generated by alum-adjuvanted vaccines.

## Graphical Abstract

**Graphical Abstract:**
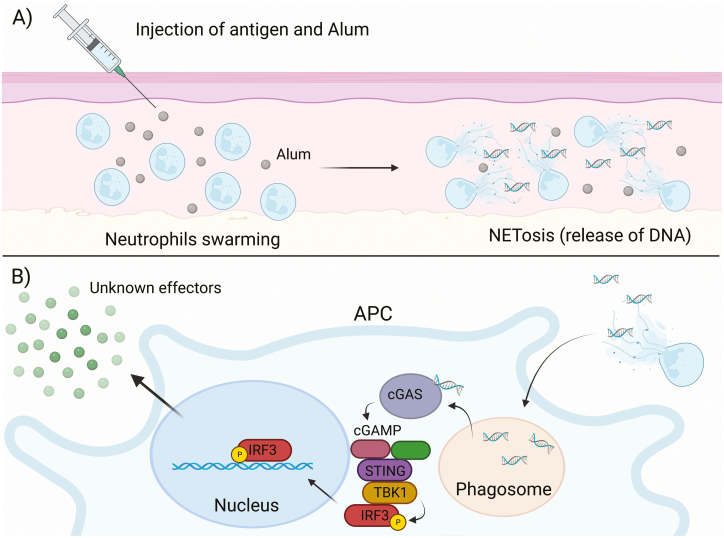

## Introduction

Vaccination has proven to be the greatest public health innovation in history [1, 2]. Countless lives across the globe have been saved as numerous infectious diseases, which were once devastating have become vaccine-preventable [1, 3]. Vaccines share the common property of stimulating antigen-specific, immunological memory to generate long-lasting immunity [4, 5]. However, vaccines take a variety of different forms to achieve this goal [3, 6]. Vaccine platform technologies have traditionally included whole killed pathogen, live-attenuated, or subunit vaccines [7–10] (Table 1). More recently, novel platform technologies, such as RNA vaccines and adenovirus-vectored vaccines, have been applied in the SARS-CoV-2 pandemic response [11, 12].

**Table 1:** Types of platform technologies currently used in human vaccine production

	Type	Form of vaccine antigen (protein)
Whole vaccines	Inactivated	Killed pathogens
	Live-attenuated	Weakened pathogens
Component vaccines	Protein subunit	Proteins isolated from pathogens
	Conjugate	Pathogen surface carbohydrates linked to a carrier protein
	Recombinant subunit	Pathogen proteins produced by an expression system by genetic engineering
	DNA/RNA based	Nucleic acids encoding pathogen proteins.
	Viral vectors	Heterologous virus containing genes encoding pathogen proteins.

Progress in vaccine development has mainly been focussed on refinement of vaccine components, with the goal of identifying the protective antigen(s) within a pathogen and using these to induce immunity [13]. Bioinformatics-led improvements in this approach, such as reverse and structural vaccinology, are very much the state of the art in vaccine development [14, 15]. These allow prediction and design of vaccine antigens without requiring access to the infectious agent, improving production, and enhancing vaccine safety profiles [15, 16].

**Figure 1:**
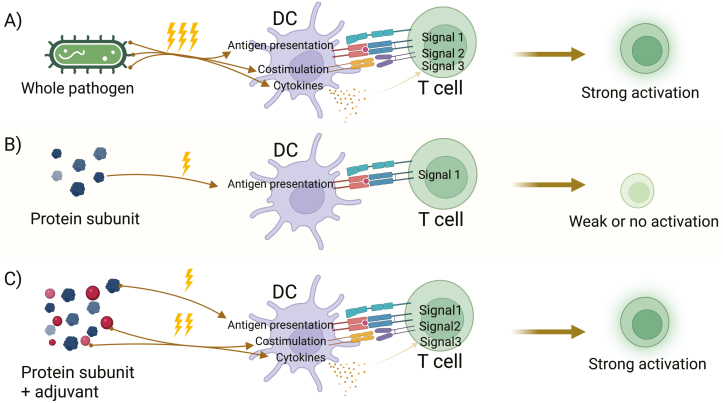
Refined vaccines have a reduced ability to stimulate immune responses, which can be restored by the addition of adjuvants. (a) Attenuated or killed pathogen vaccines contain a variety of PAMPs that can stimulate DCs resulting in strong T cell responses. (b) Dendritic cells that take up purified protein subunit vaccines can provide cognate antigen signals to T cells, but lack PAMPs and therefore fail to induce the costimulatory and cytokine signalling required for strong T cell responses. (c) The addition of adjuvants to protein subunit vaccines can restore the costimulatory and cytokine signals, resulting in stronger activation of T cells. Created with BioRender.com.

However, removing pathogen components from vaccines has had the unwanted effect of making them less effective in producing strong and durable protective immune responses [17–19] (Figure 1).

**Figure 2:**
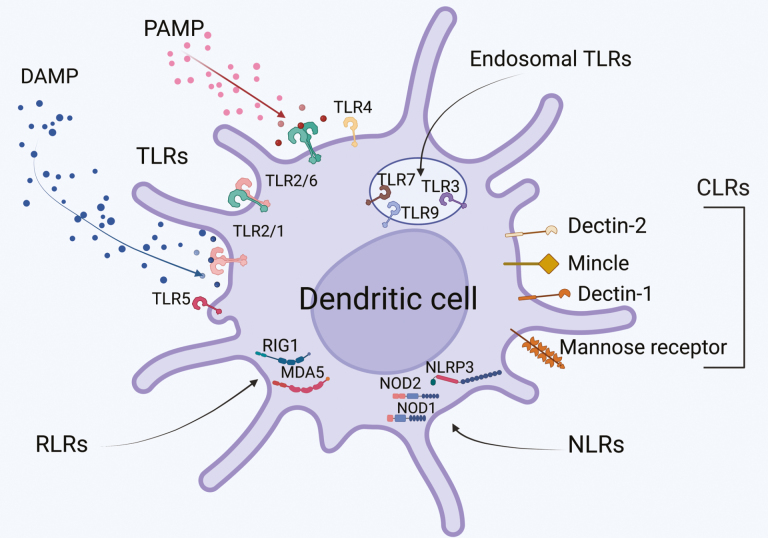
PRRs sense pathogen and danger signals, leading to Dendritic cell activation. Pathogen or Danger-associated molecular patterns (PAMPs or DAMPs, respectively) are recognised by a constellation of pattern recognition receptors (PRRs) on the surface or inside DCs. These PRRs are also the targets of vaccine adjuvants, which trigger PRRs to activate the DCs to increase antigen presentation via MHC, induce costimulatory molecule expression and drive cytokine production to enhance the activation of vaccine specific T cells. Created with BioRender.com.

Live-attenuated or whole-killed pathogen vaccines have intrinsic immunostimulators, in the form of pathogen-associated molecular patterns (or PAMPs) which are recognised by pattern recognition receptors (PRRs, Figure 2) [20]. The absence of PAMPs, such as Toll-like Receptor (TLR) agonists, in subunit vaccines, may not only contribute to the improved safety profile of the product but also result in a reduction or lack of immunogenicity [21]. In vaccine development, immunogenicity can be restored by the addition of an exogenous adjuvant (Figure 1) [19, 22–24]. One of the most ubiquitous adjuvants and the only adjuvant licensed for use in humans until the late 1990s has been the aluminium salts, more generally known as alum (Table 2) [25–27].

**Table 2:** : Alum-adjuvanted subunit vaccines in routine use in the UK

	Disease	Vaccine examples
Subunit	Diphtheria,Tetanus, Pertussis (DTP)	Infanrix hexa, Boostrix, Adacel
Subunit (conjugate)	Pneumococcal	Prevenar 13, Synflorix
	Haemophilus influenzae B	Pentacel
Recombinant subunit	Human Papilloma virus	Cervarix, Gardasil 9
	Hepatitis B	Recombivax, Engerix-B
	Meningococcal B	Bexsero

Surprisingly, despite its common use in vaccines for almost 100 years, the exact mechanism by which alum activates the adaptive immune response remains under debate. The widely accepted ‘depot effect’ theory of alum adjuvant activity was first proposed in 1925 and remained relatively unchallenged and unexplained until recently [28]. More recent studies have proposed a role for the NOD-like receptor (NLR) family, pyrin domain containing 3 (NLRP3) inflammasome, whereas others suggest indirect mechanisms where alum-induced cell death triggers DNA sensing pathways [29, 30].

These examples highlight the increasing interest in identifying alum’s mechanism of action, with the outcomes of improving adjuvant design or suggesting new combinations of adjuvants. The aim of this article is to critically review the current studies on the mechanism of action of alum, with the aim to define how this information can be used in rational adjuvant selection and vaccine design in the future.

## The depot effect

Glenny and Pope first theorised that alum’s adjuvant activity can be explained by a depot effect, after their observation of improved vaccine-induced immune responses when administering diphtheria toxin precipitated onto alum [31, 32]. Their theory was that the persisting inflammation caused by alum at the injection site, which they called a depot, caused the slower release of the antigen than if it were administered alone. The persistence of the antigen would therefore prolong immune stimulation and/or inflammation, and this results in the enhanced humoral and cellular responses observed [33]. In a later study of the persistence of diphtheria toxoid in rodents when administered with alum, Glenny *et al.* found that, in comparison with toxoid alone, toxoid plus alum persisted far longer [34].

However, more recent work by Hutchison *et al.* demonstrated that the alum injection site is not required for adjuvant activity beyond the first 2 h of injection, contradicting the depot effect theory [35]. This work demonstrated that the kinetics of antigen uptake and presentation by antigen presenting cells followed a defined sequence that was unchanged in the presence or absence of alum, or with a soluble TLR9 ligand (CpG-ODN 1826). Furthermore, it was observed that removal of the antigen plus alum injection site 2 h after administration, had no effect on antigen-specific T cell responses, T cell IL-4 production, primary or memory antibody responses, or antigen presentation by B cells and conventional dendritic cells (DCs). This would suggest that the key events that initiate the adjuvant activity of alum occur within 2 h of administration and is, again, contradictory to the theorised requirement for a long-lasting alum depot. This work is consistent with studies examining adsorption and desorption of antigens to alum for adjuvant activity [27, 36]. These show that antigens are rapidly displaced from alum in complex protein solutions that mimic tissue interstitial fluid [37, 38]. Furthermore, tight or covalent binding of antigen to alum by a process of ligand exchange acts to prevent release of antigen from the injection site and reduces the immunogenicity of vaccine formulations [39, 40]. In contrast, there may be a role for the antigen depot in affinity maturation of the B cell response to engineered antigens. Antigens modified to undergo tight binding to alum underwent slow release from the injection site and resulted in an increase in B cell activation, germinal centre formation, and long-lived plasma cell responses compared with unmodified antigen adsorbed to alum [41]. While these studies demonstrate that slow release of an antigen may contribute to enhanced B cell responses, it does not explain the mechanism of action of alum with conventionally adsorbed vaccine antigens. More likely, the studies above suggest that alum has an active impact on immune system activation, in a similar fashion to a PAMP.

## The role of the NLRP3 inflammasome

Despite these observations, previous work had demonstrated that alum could not directly activate mouse bone marrow DC *in vitro* culture [42]. Furthermore, mice deficient in TLR signalling via gene knockout of MyD88 and TIR-domain-containing adaptor protein inducing IFNβ (TRIF), maintained alum adjuvant activity *in vivo* [43]. Thus, direct activation of the immune system seemed unlikely. However, a clear requirement for DCs for alum adjuvant activity *in vivo* had been previously shown [44], and in some studies, longer incubation of DC with alum for more than 48 h induced DCs with activation signatures in human PBMC, which may have resulted from responses to alum-induced cell death in culture [45, 46].

Increasing interest in immune activation via endogenously DAMPs identified the role of the NLRP3 inflammasome in immune activation, for example, by crystalline uric acid [47]. A role for the NLRP3 inflammasome in the adjuvant activity of alum was first proposed by Eisenbarth *et al.* who observed that alum and LPS, though not alum alone, could activate Capase-1 in a NLRP3 and ASC (apoptosis-associated speck-like protein containing a caspase recruitment domain) dependent fashion in bone marrow macrophages and DCs. The requirement for LPS was also reflected in IL-1β, IL-18, and IL-33 cytokine production [48]. Studies in knockout mice demonstrated that antigen-specific IgG1 responses were dependent on NLRP3, ASC, and Caspase 1 [48]. Prior to this observation, it was known that alum stimulates caspase 1 and, consequently, the production of proinflammatory cytokines, IL-1β, and IL-18 [49]. Eisenbarth *et al.* reported that all three NLRP3 inflammasome components were necessary for the production of these cytokines in response to alum, and for the development of antibody responses, concluding that the inflammasome was required for alum’s adjuvant activity. Whether alum activates the inflammasome directly or indirectly remained unclear. The presence of alum results in increased uric acid concentrations at the site of administration thought to be due to alum-induced cytotoxicity [44]. Uric acid is an endogenous DAMP that, at high concentrations can form monosodium urate crystals, leads to the activation of the NLRP3 inflammasome [50]. The adjuvant activity of alum could be abolished by treatment of mice with uricase, suggesting an indirect mechanism of activation [44]. In contrast, Hornung demonstrated alum could activate the inflammasome in human PBMCs, independently of uric acid crystals through a mechanism of lysosome destabilisation [51].

A limitation of all the *in vitro* studies above is the requirement for a priming signal (generally LPS) to allow inflammasome-dependent cytokine production in response to alum [22]. Furthermore, the requirement of the NLRP3 inflammasome for alum’s adjuvant activity *in vivo* has become controversial. The work of Franchi and Núñez observed that antibody production in response to alum did not require the NLRP3 inflammasome [30]. McKee *et al.* have also demonstrated that alum’s adjuvant activity can occur independently of the NLRP3 inflammasome, finding that humoral (antigen-specific IgG1) and CD4 and CD8 T cell responses induced by alum were unaffected by NLRP3 and caspase-1-deficiency [50]. In addition, they observed that alum was detected by both macrophages and mast cells, and that this resulted in an influx of eosinophils, neutrophils, DCs and monocytes, and the production of numerous chemokines. This inflammatory response to alum was shown to occur independently of caspase-1, a necessary component of the NLRP3 inflammasome.

Altogether, this work suggests that, if alum does rely on the NLRP3 inflammasome for any part of its adjuvant activity, the requirement for the inflammasome is variable. The reason for this may be due to differences in the experimental conditions of these studies, or it may well be that while alum activates the inflammasome, it does not play a role in adjuvant activity [50].

## DNA sensing and NETosis

Given that neither the depot effect nor the NLRP3 inflammasome fully explain alum’s adjuvant activity, researchers have pursued new immunological directions. Double stranded, host DNA accumulates at the alum injection site and can also act as an endogenous DAMP [52]. Extracellular DNA was shown to act as an adjuvant, and may contribute to the immune response to alum, as treatment with DNase (which would degrade extracellular DNA) in mice immunised with antigen plus alum reduced antigen presentation and CD4+ T cell responses [53] and antigen-specific IgE production [52]. While initial studies suggested that host DNA acts to enhance the arrival of antigen presenting cells in the draining lymph node [52], other studies indicate this is not the case, and DNA may act directly on antigen presenting cells in the lymph node to enhance interactions with T cells [53]. As a note of caution, the effects of DNase may be artefactual as they been shown to be susceptible to contamination with proteases, which may also digest antigen [54]. Nevertheless, studies employing gene knockout approaches to determine the mechanisms by which alum induces the release of host DNA and on which cells it has this effect, are not susceptible to this potential artefact [55, 56].

**Figure 3:**
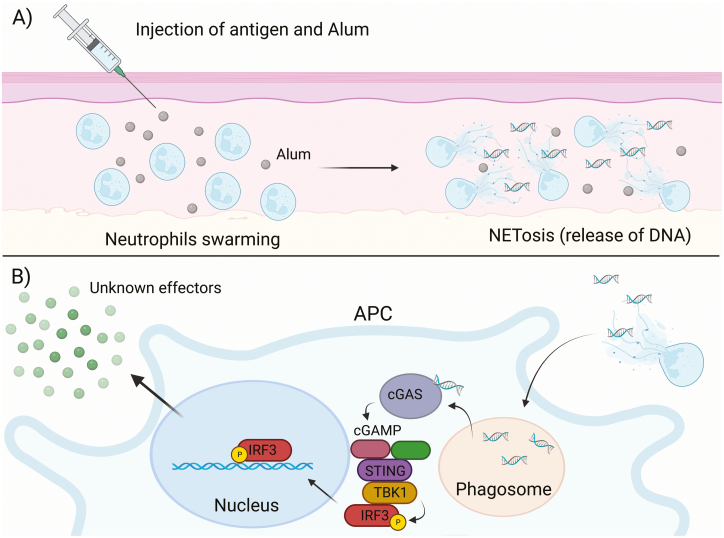
Alum induces rapid neutrophil swarming and NETosis that activates the cGAS-STING signalling pathway and confers its adjuvant activity. (a) Neutrophils are rapidly recruited to the site of injection and form neutrophil swarms around the deposited alum. The presence of alum induces the recruited neutrophils to undergo rapid NETosis that releases host DNA into the extracellular space. (b) Antigen presenting cells engulf the released NETs and the host DNA that was expelled with them. After exiting the phagosome (facilitated by the neutrophil elastase protein), the DNA is recognised in the cytosol by cyclic GMP-AMP (cGAMP) synthase (cGAS) receptor. cGAS generates cGAMP, which recruits the adaptor protein, stimulator of interferon (IFN) genes (STING). STING recruits TANK-binding kinase 1 (TBK1) which then recruits and phosphorylates IFN regulatory factor 3 (IRF3). Although IRF3 is a known transcription factor responsible for type I IFN production, it is unlikely that alum adjuvant function is dependent of these cytokines, so the final effector responses induced by IRF3 remains unclear. Created with BioRender.com.

Stephen *et al.* found that rapid recruitment of neutrophils within 2 h of alum administration produced neutrophil swarms around the alum deposits [56]. This is consistent with previous findings where the events that confer alum’s adjuvant activity occur within 2 h of administration [35]. The researchers also observed that, following the rapid influx of neutrophils to the site of alum administration, alum induces NETosis (a neutrophil-specific form of cell death involving the formation and release of neutrophil extracellular traps - NETs) by those neutrophils and that this process is PAD4-dependent. NETs are primarily composed of host DNA and are released by neutrophils during the innate immune response for the main purpose of trapping pathogens [57]. The NETs and any trapped microbes may then be phagocytosed by APCs, such as macrophages (Figure 3). Stephen *et al.* also demonstrated that PAD4-deficient mice, whose neutrophils are incapable of undergoing NETosis, had diminished cellular and humoral immune responses to alum; thus, indicating that the adjuvant activity of alum is partially mediated by NETosis [56].

Double-stranded DNA, such as the host DNA released in NETs, acts as a DAMP when recognised by PRRs, primarily TLRs [58]. While many pathogen-derived adjuvants induce an immune response via TLRs, as mentioned earlier, alum’s adjuvant activity occurs independently of TLR signalling [43]. Sun *et al.* found that cGAS, a cytosolic PRR, is responsible for recognition of double-stranded DNA in the cytosol [59]. cGAS induces type I interferon (IFN) production through the cGAS- stimulator of IFN genes (STING) pathway.

Apel *et al.* investigated whether double-stranded DNA from NETs could be recognised by cGAS and activate production of type I IFNs, finding that this was the case when NETs were phagocytosed by macrophages both *in vitro* and *in vivo* [60]. They observed that the phagocytosed NETs were released into the cytosol of macrophages, seemingly facilitated by the neutrophil elastase (NE) protein (a neutrophil serine protease present in NETs alongside host DNA), where they could be detected by the cytosolic cGAS receptors. Following cGAS activation, cGAMP is generated and recruits the STING adaptor protein. STING recruits TANK-binding kinase 1, which then recruits and phosphorylates IFN regulatory factor 3 (IRF3). Finally, IRF3 a transcription factor, activates type I IFN gene expression in the nucleus [61].

The exact roles that the components of the cGAS-STING signalling pathway play in eliciting the alum-induced adaptive immune response are still not entirely clear. Marichal *et al.* demonstrated the requirement for IRF3 in alum-induced Th2 responses and IgE production [52]. This seems unlikely to be dependent on type 1 IFN responses that typically act to reduce Th2 responses [62]. In support, McKee *et al.* determined that alum adjuvant activity was not dependent on type I IFNs, but did observe that cellular and humoral responses to alum were reduced in STING-deficient mice [53]. In conclusion, host DNA, most likely sensed via cGAS and signalled via STING/TBK/IRF3, plays an important role in alum adjuvant activity. However, the key effector responses triggered by these signalling pathways to mediate adjuvant activity, remain to be identified.

## Discussion

While the theory of the depot effect has long been a popular one, and a role for the NLRP3 inflammasome has been hotly debated, the complete mechanism by which alum confers its adjuvant activity has never been elucidated. However, recent evidence has indicated very strongly that rapid neutrophil recruitment and NETosis, DNA sensing by the cytosolic PRR cGAS, and signalling through the adaptor protein STING are the major contributors to the adjuvant activity of alum.

Based on the work of both Hutchison *et al.* , it is quite clear that the events that determine the immune response to alum, which is characterised as a Th2-type response, occur within the first 2 h of alum administration [35]. Stephen *et al.’s* work is consistent with this, finding that neutrophil swarming and NETosis occurs very rapidly within those 2 h [56]. Apel *et al.’s* work also corroborates this, as they have identified that DNA produced via NETosis is recognised by the cGAS-STING pathway, which produces type I IFNs [60]. Although the work of McKee *et al.* shows that the production of type I IFNs is not required for the adjuvant activity of alum, their findings do coincide with the overall theory that NETosis is responsible for adjuvant activity as they have identified extracellular host DNA and STING as necessary [53]. However, the production of type I IFNs in response to NETosis may contribute to inflammatory responses induced by alum [60].

Despite this progress, there are still many questions remaining about the mechanism of alum’s adjuvant activity. First, how does alum induce neutrophil recruitment and NETosis at the site of its administration? Neutrophils respond to the presence of alum very rapidly, which is likely to require preformed rather than transcriptional production of chemokines [56, 63]. One potential mechanism may involve formyl peptide receptor 1, which can rapidly induce the recruitment of neutrophils in response to bacterially derived formylated peptides [64]. Similar formylated peptides are also released from host mitochondria during cell death driving neutrophil swarming at the site of injection. Therefore, it is possible that, as a result of alum cytotoxicity on bystander cells or neutrophils, formylated mitochondrial proteins are quickly released by damaged cells and rapidly recruit neutrophils.

Still, this proposal does not answer the question of what causes the recruited neutrophils to so rapidly undergo NETosis. Another remaining question is the function of the STING protein in the adjuvant activity of alum. McKee *et al.* demonstrated that adjuvant activity is reliant on STING but not type I IFNs, the primary cytokine released through the cGAS-STING signalling pathway [53]. It was suggested that STING may confer alum’s adjuvant activity through an alternative pathway, such as one involving signal transducer and activator of transcription 6 as described by Chen *et al.* [65], or that the mechanism involving type I IFNs is a redundant one [53]. Finally, it remains to be determined exactly whether the NLRP3 inflammasome does play a partial role in the adjuvant activity of alum.

Determining the exact mechanism conferring alum’s adjuvant activity is motivated by two main desires. First, alum has been used in human vaccines for nearly a century, yet we still do not understand exactly how this substance functions once in humans. There is a growing problem of vaccine hesitancy globally, which is fed at least in part by the spread of misinformation and lack of sufficient vaccine education [66]. Improving our understanding of how our vaccines work and being able to effectively communicate this to the public is essential to build trust in vaccines. Second, increasing our understanding of the mechanism of action of currently available adjuvants is crucial for improved adjuvant design and the development of new adjuvants in the future. Infectious diseases remain a major burden on global health and the development of novel vaccines, and adjuvants to improve their immunogenicity, to combat this will continue to be a priority in health research.

## Abbreviations

ASC: apoptosis-associated speck-like protein containing a caspase recruitment domain
cGAS: cyclic GMP-AMP
DAMP: danger-associated molecular pattern
IRF3: IFN regulatory factor 3
LPS: lipopolysaccharide
NLR: NOD-like receptor
NLRP3: NLR pyrin domain containing 3
PAD4: peptidyl arginine deiminase
PAMPs: pathogen-associated molecular patterns
PBMC: peripheral blood mononuclear cell
PRRs: pattern recognition receptors
STING: stimulator of interferon genes
TBK1: TANK-binding kinase 1
TLR: toll-like receptors
TRIF: TIR-domain-containing adaptor protein inducing IFNβ
